# The Bengal Dispensatory and Companion to the Pharmacopœia, Chiefly Compiled from the Works of Roxburgh, Wallich, Ainslie, Wight and Arnot, Royle, Pereira, Lindley, Richard, Feé, and Including the Results of Numerous Special Experiments

**Published:** 1843-10

**Authors:** 


### Art. VI.

1. *The Bengal Dispensatory and Companion to the Pharmacopœia, chiefly compiled from the works of Roxburgh, Wallich, Ainslie, Wight and Arnot, Royle, Pereira, Lindley, Richard, Feé, and including the results of numerous special experiments.* By W. B. O'Shaughnessy, m.d., Assistant Surgeon to the Bengal Army, &c. &c., Professor of Chemistry and Materia Medica in the Medical College of Calcutta. *Published by order of the Bengal Government.—Calcutta,* 1842. 8vo, pp. 794.

2. *Elements of Materia Medica and Therapeutics, including the recent discoveries and analysis of Medicines.* By Anthony Todd Thomson, m.d. f.l.s. &c. Third Edition, enlarged and improved.—*London,* 1843. 8vo, pp. 1232.

3. *The Elements of Materia Medica and Therapeutics.* By Jonathan Pereira, m.d. f.r.s. & l.s. &c. Second Edition.—*London,* 1842. Two Vols. 8vo, pp. 1926.

We have comprised these three works under one head, because they all relate to one general subject. We shall, however, review each one separately, and content ourselves with a very brief notice of the last two on our list, as they are only new editions of works already noticed by us.

### I. DR. O'SHAUGHNESSY'S BENGAL DISPENSATORY.

Several years ago the Governor-general of India (Lord Auckland), in council, appointed a Committee to inquire into various subjects connected with the state of the materia medica in British India, and on the expediency of compiling a Pharmacopœia for Bengal and Upper India. The committee consisted of six persons, viz. Drs. W. Jackson, J. Ranken, Pearson, and W. B. O'Shaughnessy, and Messrs. Bramley and James Prinsep. But long before these gentlemen could accomplish the important labours they each most zealously undertook, they were scattered by death, ill-health, and the casualties of service; and to Dr. O'Shaughnessy was intrusted the task of reporting on the subject of the proposed pharmacopœia. This gentleman at that time held the situation of Professor of Chemistry and Materia Medica in the Medical College at Calcutta, and was in every respect ably qualified to fulfil the task imposed on him.

In due time the report made its appearance. It consisted of some observations on the nature and uses of the pharmacopœias in general; a review of the leading arguments for the preparation of a pharmacopœia for Bengal; and notice of some of the circumstances requisite to render it of the widest and readiest utility; and lastly, a sketch of the character and extent of investigation which must indispensably precede the compilation.

In alluding to the causes which have led to the compilation of so many pharmacopœias, the report glances at the remarkable varieties of raw material, from which in different countries one and the same medicinal agent has to be prepared.

"Take potash and its compounds for example—obtained in Canada by the burning of the forest timber; in Ireland from the fern; in France, Italy, and along the Rhenish wine districts, from cream of Tartar; in India, with most economy, from nitre; in each case a totally different process is required for its extraction, purification, and adaptation to medicinal use. Soda gives us another and a striking instance.—The Spaniard obtains it most easily from barilla by mere washing; the Scot from kelp by a much more laborious and unproductive process; in France, and latterly in England, it is prepared from common salt (muriate of soda), which is first decomposed by sulphuric acid, and then the resulting sulphate of soda subjected to another process, which changes it into carbonate. In Bengal and Mysore, again, the soil, in many places, is so impregnated with the alkali, that incineration and washing extract from 35 to 50 per cent. of fine soda." (Introduction, p. vii.)

In considering the necessity and probable degree of utility of compiling a Pharmacopœia for Bengal, the report alludes to the high prices of all medicinal preparations sold by the private European establishments in Calcutta, Cawnpore, and Meerut, the only localities in Bengal or Upper India where European apothecaries have settled.

"Little competition existing, the prices of the most indispensable articles of medicine are fixed at such a rate that rich natives will not, the humbler cannot, avail themselves of the remedies which medical science has pointed out. . . . . . . . We are prepared to show, that in the houses of well, nay highly, educated natives, medicines of every kind may be retailed in Calcutta, and throughout India, at lower prices than even those of the London general practitioners, who charge at least 200 per cent. less than their Calcutta brethren, and give the patient the benefit of their attendance besides." (Ib. p. ix.)

These extracts show the highly interesting nature of the report, the suggestions of which were ordered by the India government to be carried into effect; and Dr. O'Shaughnessy was directed to devote himself to the task of compiling not only a pharmacopœia but a dispensatory of general materia medica. It now becomes our duty to examine the latter work, the title of which stands at the head of this article.

The work in question treats of a great variety of subjects, too many in fact to be properly investigated in a book restricted to one volume of 800 not very closely-printed pages. It embraces instructions on pharmaceutical manipulations; on the mode of taking specific gravities; of making ordinary meteorological observations, (explanatory details being given under each head;) an outline of chemistry for the guidance of the teachers of native apothecaries; a grammar of botany, constituting a systematised glossary of the terms used in botanical descriptions; a brief account of the mode of action of the several therapeutical classes of remedial agents; the vegetable materia medica arranged in the natural system; the animal materia medica; miscellaneous inorganic substances; and, lastly, an appendix on the manufactory of an improved pottery in Bengal; on the investigation and treatment of poisoning; with meteorological tables and notices of miscellaneous subjects.

We greatly regret that Dr. O'Shaughnessy did not think it expedient to devote the whole of his restricted volume to the proper and legitimate objects of a dispensatory; instead of introducing into it so great a variety of topics, many of which are quite foreign to a work on pharmacology. What connexion, for example, has the manufacture of an improved pottery with a dispensatory? Moreover the attempt to give epitomes of meteorology, of pharmacy, of chemistry, and of botany in a work of this kind, must, as far as practical utility is concerned, prove a failure; the details on each subject being too meager and imperfect to be useful; while their introduction is positively objectionable on the ground that they occupy that space which ought to be devoted to other more appropriate objects of inquiry. Accordingly, Drs. Wood and Bache in the United States Dispensatory, and Dr. Christison in the Edinburgh Dispensatory, have thought it best not to attempt any epitome of pharmacy which had been given by the compilers of previous dispensatories; and we wish that Dr. O'Shaughnessy had adopted the same plan.

We proceed now to a more pleasing duty, that of laying before our readers some of the valuable information, relating to the Indian materia medica, which is contained in Dr. O'Shaughnessy's work; and in doing this we shall, for the most part, select those substances which from being used in Europe, are most likely to interest our readers.

*Opium.* Dr. O'Shaughnessy gives the following information respecting the mode of preparing Indian opium.

"Towards the end of March when the plant is nearly ripe the bleeding of the capsules takes place. This operation is performed in the evening, and consists of tracing four parallel lines with a sharp knife or shell, penetrating the epicarp and sarcocarp of the capsule; a milky juice immediately exudes, and its flow is promoted by the deposition of dew during the night; each set of incisions yields on an average one grain of opium. This is removed the ensuing morning, and the incisions repeated as long as any milk flows. The whole of the opium collected daily, and which is necessarily mingled with variable proportions of dew, is mixed together in earthern or wooden vessels. Should the quantity of dew have been excessive, a partial solution of the opium occurs, and an exudation of drops of a black, shining liquid (Pasewa) occurs on the surface of the opium. This pasewa contains many of the active principles of the drug, and should be mixed with it again to secure the uniform power of the mass. The proportion of pasewa is often much increased by the fraudulent admixture of water by the growers, in hope of having their opium purchased by the gross weight.

"When brought to the factories the opium is usually of the consistence of 64 to 68, that is, containing that proportion of solid matter per 100. It is then placed in large tanks of brickwork, or in wooden vats, with the upper surface exposed to the air. From these reservoirs portions are daily removed and exposed on wooden trays three inches deep by four feet long and two broad; evaporation takes place under such circumstances with tolerable rapidity, and the opium eventually reaches the consistence of 70—the standard of the factory for the Chinese investment.

"The degree of inspissation is estimated on the purchase of the drug, as well as during its subsequent evaporation, by drying average portions of 100 grains each, on a metallic table heated to 208° by steam.

"A sufficient quantity of opium, of average quality, is permitted to attain the consistence of 80°, and in that state is issued under the name of "Abkaree" to the licensed dealers of the bazars. Lastly, the finest opium which can be procured is set apart for medical purposes, and allowed to lose by evaporation nearly the entire of its water.

"The *Chinese investment opium* is of a rich chesnut colour, translucent at the edges of thin masses, tears into portions with ragged margins; its stucture is granular, its taste hot and bitter, its smell rich, peculiar, and agreeable; if a small portion be rubbed between the finger and thumb for a few seconds it draws out into long colourless threads, and from the number, fineness, and tenacity of these threads the Chinese form their first estimate of the value of the drug.

The "*abkaree*," or Bengal bazar opium is more solid than the Chinese investment drug, of a darker colour, and often of a heavy ascescent smell.

For the Chinese investment the opium is made into cakes, each containing four pounds, and enveloped in a case made of the petals of the poppy, and agglutinated with a paste made of opium and water, (Lewa.) The abakaree opium is also vended in balls weighing two pounds, covered by strips of a coarse kind of silk cemented by a mucilage of gum. The medicinal opium is solid, brittle in the cold season, of a brown colour, and fine smell. The medicinal drug is packed with great care in four pound and two pound squares, covered with layers of talc, and further defended by a case of brown wax, half an inch in thickness." (pp. 172-4.)

The quantity of pure morphia present in opium varies according to the kind:

| | |
|---|---|
| "Bengal investment opium contains . . . . . | 2½ per 100.* |
| Medicinal opium . . . . . . . . . . | 4 |
| Malwah ditto . . . . . . . . . . | 6 |
| Garden opium, Patna . . . . . . . . | 10½ |
| Turkey opium . . . . . . . . . . | 9 |
| Smyrna opium . . . . . . . . . . | 10½" (p. 176.) |

*Opium eating.* It is a common but an erroneous notion entertained in this country, that the habitual use of opium, whether by chewing or

* "The official returns by the opium examiner of Calcutta for the last four years show the following per centage of *morphia* in a 'tolerably pure' state:

| 1836. | 1837. | 1838. | 1839." |
|---|---|---|---|
| 3·7 | 3·8 | 4·0 | 3·9 |
| 4·1 | 4·1 | 3·9 | 4·0 |
| 3·6 | 4·1 | 4·0 | 3·7 |

smoking, has a direct tendency to shorten life. That its excessive employment is attended with this effect must be admitted; but that its moderate use has any such tendency we deny. Several years ago Dr. Christison gave abstracts of eleven cases of opium-eating from which it appeared that the popular notion of the practice being injurious to health and shortening life was erroneous; and Dr. O'Shaughnessy in speaking of opium-eating, observes that it has not been "ascertained that this habit has a direct tendency to shorten life; on the contrary, the longevity of opium-eaters is in many parts of the East of proverbial notoriety."

*Opium-smoking.* On this subject Dr. O'Shaughnessy observes:

"The vice of opium-smoking is now so prevalent in Calcutta, not only among the Chinese, but the degraded and depraved of every sect and nation, that we have had many opportunities of witnessing the results to which it leads.

"Stupor, reverie, and voluptuous listlessness are the immediate effects produced. In this state the individual can be at once and easily aroused to exertion or business. No sickness, constipation, or any other functional disturbance supervenes on each indulgence; gradually, however, the appetite diminishes, the bowels become irregular, emaciation takes place, sexual tendencies are destroyed, and premature old age speedily comes on. This we admit is an extreme case; when the habit is but moderately followed it appears to occasion no greater evil than the proportionate indulgence in wine or other spirituous liquors." (pp. 180-1.)

*Anarcotina*, (*Narcotina.*) Narcotina, which Dr. O'Shaughnessy calls anarcotina, is devoid of all narcotic properties and is capable of arresting the paroxysms of intermittent and remittent fevers.*

*Gurjun balsam.* As gurjun balsam may be employed as a cheap substitute for balsam of copaiba, and as a very large quantity of it was, a few years back, brought to this country and is we believe yet unsold, it may not be uninteresting to extract a part of Dr. O'Shaughnessy's notice of it.

It is the produce of the *dipterocarpus lævis*, a large tree growing in Chittagong, Pegu, Assam, Tippera, &c.

"To procure the balsam, Dr. Roxburgh informs us a large notch is cut in the trunk of the tree near the earth, where a fire is kept up till the wound is charred, soon after which the liquid begins to ooze out; a small gutter is cut in the wood to conduct the liquid to a receiver. The average produce is said to be 40 gallons in each season, during which it is necessary to cut of the charred surface from time to time, and burn it afresh. The process is performed during the cold season.

"The gurjun balsam varies in consistence from that of thick honey to a light oily liquid. The colour of a fine specimen of thick gurjun obtained from Captain Jenkins of Assam, was a pale grey; specimens sent from Rangoon by Mr. Spier were a light brown. As found in the bazar this substance generally occurs as a brown, oily looking, semitransparent liquid, in odour strongly resembling a mixture of balsam of copaiba with a small portion of naphtha.....

"The close resemblance in the physical and chemical properties of this gurjun and copaiba balsam led to the institution of an extensive set of experiments by the Editor on the medicinal effects of the former in the treatment of gonorrhœa. The results, which have been laid before the profession, and which have been confirmed by trials made by other practitioners, seem perfectly conclusive that in the treatment of gonorrhœa, gleet, and similar affections of the urinary organs the essential oil of gurjun is nearly equal in efficacy to the South American drug.

"The essential oil may be given in ten to thirty drop doses in mucilage, milk, rice-water, or thin gruel, and repeated thrice, or still more frequently daily. It

* See an Analysis of Dr. O'Shaughnessy's paper on Narcotine as a substitute for Quinine in Intermittent Fever in the Brit. and For. Med. Rev., vol. VIII. p. 263.

generally causes a sensation of warmth at the epigastrium, eructations, and sometimes slight purging. It communicates a strong smell of turpentine to the urine, which it increases remarkably in quantity. Some obstinate cases of chronic gonorrhœa and gleet, which had long resisted copaiba and cubebs, have been cured by this remedy in the course of the experiments alluded to." (pp. 223-4.)

*Indian hemp.* The plant which yields this is the *Cannabis Indica* of botanists, and is probably a variety only of the common hemp, or *Cannabis sativa.* A very full account of the effects and uses of this plant is contained in the Dispensatory; but as we have already given (see Brit. and For. Rev., vol. X. p. 225) a very full analysis of Dr. O'Shaughnessy's pamphlet on this subject, it will be unnecessary to enter further into it, except to observe that subsequent trials with this plant in this country have confirmed neither its asserted activity nor its therapeutical efficacy. Whether this be owing to the preparations having undergone some deterioration in their passage, or to the comparative phlegmatic temperament of the English, we know not. We understand that in a recent case of tetanus in the London Hospital, the extract prepared by Mr. Squire, of Oxford Street, from plants brought from India by Dr. O'Shaughnessy, was given in very large doses but without any apparent alleviation of the symptoms; and in a few days the man died. The case, which was originally under Mr. Luke's care, was transferred by him to Dr. Pereira; and the exhibition and effects of the medicine were watched by Dr. O'Shaughnessy. In this case the hemp acted as a soporific, and produced a slight tendency to inebriation. There was also an approach to the cataleptic condition, for when the arm was very gently and slowly raised it sometimes remained, when left to itself, for a few minutes in the same posture. During its use the pupils were contracted; but in most cases dilatation follows the use of hemp. The medicine had also a complete trial, in a case of idiopathic tetanus, at Guy's Hospital, by Dr. Babington. It was used both externally and internally, but without any manifest effect on the system or the disease.

*Grass oil of Nemaur* (Roosa-ke-tel, *Hind.*) As this oil is met with in the London shops under the name of *Oil of spikenard*, or *Oil of Nard*, while its origin is but little known, we subjoin Dr. O'Shaughnessy's account of it; premising that the grass which yields it has been denominated by Professor Royle the *Andropogon calamus aromaticus*, and is probably identical with the "*Sweet calamus*," and the "*Sweet cane from a far country*" of Scripture,—the κάλαμος ἀρωματικὸς of the Greeks.

"This valuable oil was first brought to the notice of the profession by Dr. Maxwell in 1824, and was further described by Dr. Forsyth in 1826.

"The oil is obtained from the grass by distillation; 250 to 300 small bundles of the grass are placed in a boiler, covered with water, and distilled. About a seer of oil is obtained in the receiver. Dr. Forsyth describes it as volatile, extremely pungent, of light straw colour, very transparent, with a peculiar rich and agreeable odour.

"Dr. Forsyth adds, that it is very highly esteemed by the wealthy natives for the cure of rheumatism, especially that of the chronic kind; two drachms of the diluted oil are rubbed over the pained part in the heat of the sun or before a fire twice daily. It causes a strong sensation of heat or pricking, lasting for two hours or longer. The natives also regard it as an efficacious remedy in slight colds. They anoint the soles of the feet with the oil, and it is stated that slight diaphoresis is thus produced.—See Trans. Med. and Phys. Soc. iii. p. 216." (pp. 639-40.)

*Nux-vomica bark* (*Kuchila.*) It is now well known that the bark called by European druggists "*false angustura*," is the bark of the *Strychnos nux vomica.* Though its nature was long suspected, we are indebted to Dr. O'Shaughnessy for first clearly determining it. It appears also from his statement that the same bark is commonly sold in Calcutta under the name of "*Rohun*," and substituted for the harmless bark of *Soymida febrifuga* (Swietenia febrifuga.)

"In vol. iv. Trans. Med. Phys. Society of Calcutta there is a paper by Mr. Piddington on the rohuna bark, and the sulphate of its bitter principle. Mr. Piddington stated that he had applied to the bark the process described by Paris (Pharmacologia, vol. ii.) for preparing sulphate of quinine, and with perfect success. He presented to the society a small specimen of the alleged sulphate of rohuna, and described in full detail the process of its preparation. In 1836, however, it was ascertained by the editor of this work that the salt prepared by Mr. Piddington was sulphate of *Brucine*, and that the bark he operated on was that of the nux-vomica tree. On inquiry in the bazars it was found that in several shops the nux-vomica bark was sold for the rohun." (p. 248.)

We have been informed that a large quantity of the supposed "sulphate of rohuna" (in reality sulphate of brucine or strychnine,) had been prepared at Calcutta for the use of the military hospitals of India, as a substitute for the sulphate of quinine; and that some of it had actually been sent off for use. Fortunately, however, Dr. O'Shaughnessy discovered its true nature in sufficient time to prevent the occurrence of any accident.

*Aloes.* We have been disappointed in not finding in the Dispensatory some notice of the origin of the aloes exported from Bombay, under the name of *socotorine and true hepatic aloes.*

"We are indebted to Mr. Heddle of Bombay, for three specimens of aloes, marked Socotorine, Kurachee, and Deckan.

"That marked *socotorine* is rich, brown, shining, soft, ductile, and adhesive at 82° Fahr.; of strong and not disagreeable odour, wrapped up in and including leaves. It much resembles the best Bengal opium in general appearance. Water dissolves 82 parts per 100.

"The *Kurachee aloes* is nearly black, opaque, rather vascular, structure of shelly fracture, faint smell, powder blackish, slightly adhesive to the touch. It is identical with the better kinds of Musabhir of the Bengal bazars. Water dissolves only 52 per 100.

"The *Deckan* specimen is deep brown, of earthy and slightly resinous fracture, and faint odour, powder brown. Soluble in water in proportion of 98 per 100.

"The common *bazar aloes* in Bengal is black, brittle, of semi-vitreous fracture, and too impure for medical use." (p. 666.)

*Kino.* Dr. O'Shaughnessy gives us no information as to the source of the extract called kino, imported into this country from India, and which is the produce of some part of Lower India. The concrete juice of *butea frondosa*, which he calls in one place the *Bengal kino*, and in another *Indian kino*, is a very different substance to the extract known here as kino; and we regret, therefore, that he should have given it these names, since they are calculated to lead to considerable confusion.

*Catechu.* We expected to have found in the Dispensatory a full account of the various astringent extracts brought from India, and known in European commerce as *terra japonica*, *catch*, and *catechu.* But while Guibourt describes eleven kinds, and others a still greater number of varieties, Dr. O'Shaughnessy names three only, which he calls *Bengal catechu*, *Bombay catechu*, and *Massive catechu.* It is greatly to be regretted that on this, as well as on other occasions, he should have relied on an European botanist for his pharmacographical details. We allude now to Fée, who, whatever may be his other merits, is, as a pharmacologist, inferior to Guibourt, Martius, and others. And we strongly protest against the practice of writers on Indian Materia Medica, taking their descriptions from European pharmacologists, as it leads to inextricable confusion.

With these remarks we close our notice of the Bengal Dispensatory. From the extracts we have made, our readers will be enabled to form a good idea of the nature and valuable quality of the information to be found in it. The work will be found invaluable to all European surgeons and physicians who are practising or intend to practise in India. To the educated native practitioner it must prove of the highest service. While, lastly, to the scientific European pharmacologist it is an indispensable work.

### II. Dr. Thomson's Elements of Materia Medica.

In the advertisement to the present edition, Dr. Thomson states that it "is not intended to afford minute details of the natural history and the commerce of the medicinal agents it treats of; but to supply that general information respecting the chemistry and therapeutical employment of the articles of the materia medica, which is likely to prove practically useful to students and junior practitioners of the healing art." We have strong objections to this plan, in a work chiefly appropriated to therapeutics, for the obvious reason, that the chemical and other details respecting the properties of the various substances cannot so conveniently be given as under a natural-historical or alphabetical arrangement. On this account the form of dispensatory, previously adopted by the author, was much better adapted for the purpose. Again, a work on therapeutics should be restricted to that subject and should not be overlaid with the accumulated rubbish of inert and doubtful medicines, which, however, may be very properly described elsewhere as articles of natural history, applicable to some useful purpose. Such a work ought to concentrate its force upon the really active and efficient remedies.

The classification of medicines adopted by Dr. Thomson is chiefly based on that of Dr. Murray; but we went so fully into the subject, in the analysis of Dr. Paris's work in our last Number, that our limits will not permit us again to enter upon it. We may remark, however, that emetics are placed under the division of agents that act on the muscular and sanguineous systems, and cathartics under those which excite the secerning and exhalent systems. This violent separation of substances so clearly analogous if not identical in their action is absurd: all purgatives act more or less on the muscular tissue of the intestines, and thus increase the peristaltic motion of the intestines. The number of substances in the list of excitants (the first order) is enormous, and it contains agents of very doubtful pretensions, and which are allied to one another by few family resemblances. Among these may be enumerated, nux vomica, strychnia, brucia, veratria, camphor, the preparations of gold and mercury, electricity, chlorine, iodine and bromine with their combinations; besides a number of vegetable substances, not admitted into the British pharmacopœias. We admire the author's industry and research, but we question very much the propriety of uniting into one order, such a mass of heterogeneous materials. The common-place student would be quite bewildered by such a formidable array of forces; and many who, in their study of geometry, passed with ease the *pons asinorum* would here be beset with insuperable difficulties. We shall, however, let the author speak for himself.

"In prescribing any of these material agents, or in taking advantage of the mental affections in this collection of excitants, the general intention of the physician is to sustain the powers of life under diseased conditions of the frame; to rouse action when it is defective; and, finally, to imitate nature in setting up *temporary movements* in the system, resembling disease, or what has been termed *reaction*, in order to restore the balance of the circulation, and that equilibrium of all the functions which constitute health." (p. 234.)

The field indicated in this account of their action is by no means very definite, and might without much stretch of imagination, have included nearly every article of the materia medica; for all are prescribed with the direct or indirect object of restoring the circulation and the other functions of the body to their healthy standard.

*Camphor*. The following account of the various virtues of this drug affords a good specimen of the style of the work, and shows the loose and unguarded manner in which the author is apt to indulge in giving an account of the qualities of remedies:

"There can be no doubt that camphor, which forms a group by itself, although it has so close an affinity to the volatile oils, is properly placed in this class of medicinal agents. Its excitant properties are confirmed by its influence in stimulating the uterus to renewed action when its efforts have been suspended, during parturition, by the influence of opium, or by hemorrhages, or any other cause; a purpose for which it has long been successfully employed by Dr. Hamilton, the Professor of Midwifery in the University of Edinburgh. . . . . . The diversity of the action of camphor depends on the dose in which it is administered. A modification of its action is produced by combining it with some other medicines; as for example, with antimonials, nitre, and neutral purgative salts. In combination with opium and aromatics, its influence in checking the progress of gangrene and in upholding the powers of the constitution in confluent smallpox, measles, and other eruptive fevers, when these take on the typhoid character, and the eruptions recede, has been well confirmed; and in these cases its effects can only be explained by admitting its excitant properties. . . . . In summing up the properties of camphor, it may be justly stated that it partakes of the properties of many of the other classes of medicines; that it proves excitant, or sedative, or narcotic, or antispasmodic, or diuretic, according to the combinations in which it is administered and the condition of the patient at the moment of administration. In full doses, namely, from gr. x to gr. xx, given alone, in low and depressed states of the vital energy, its excitant power is displayed; in combination with antimonials and mercurials, it lowers action and operates as a sedative: with opium its narcotic powers are developed and those of the opium augmented; with salts of quina it is either excitant or antispasmodic; and with nitrate of potassa the influence of salt upon the kidneys is both ensured and increased. Its powers, in combination, of diminishing the drastic and griping powers of resinous cathartics, at the same time that it aids their purgative property, was formerly mentioned." (pp. 239-42.)

If these statements be correct camphor certainly possesses very valuable properties; but we had no conception that any modern author held such a high opinion of its therapeutic virtues. First, with regard to its power in stimulating the inactive uterus during parturition, we may remark that it may, like alcohol and other stimulating substances, occasionally prove useful, but certainly its merits in such cases are in general little esteemed by obstetrical practitioners. Does the author really believe that camphor, along with opium and aromatics, will check the progress of gangrene? We thought that the more accurate observation of the nineteenth century had almost entirely dissipated this misconception. This agent may assist in supporting the strength, during the progress of gangrene and the various eruptive fevers accompanied with a typhoid type, but, in this property, it is greatly inferior to wine or diluted alcohol, and is, moreover, attended with more risk when exhibited in large doses. We should like to know how ten to twenty grains of camphor united with any antimonial or mercurial would lower action and operate as a sedative? Is its exciting powers completely extinguished by combination? for Dr. Thomson elsewhere states (p. 296) that "sedatives are substances which directly depress the energy of the nervous system, diminishing action in animal bodies without inducing previous excitement." How do the salts of quina render it excitant or antispasmodic, when it possesses inherently these very properties?

As the treatment of fever by alcoholic fluids is a subject upon which various opinions are held, we have pleasure in quoting the following judicious remarks:

"It is, however, evident that wine and alcoholic fluids are seldom or never necessary in the early stages of fever; even in the later stages the propriety of their administration must altogether depend on circumstances and the nature of the prevailing epidemic. The nearer that the symptoms approach those of typhus, the more they are likely to prove beneficial. It is in the stage of collapse, or failure of power, denoted by a rapid, soft, intermittent pulse, the tongue tremulous when protruded, low muttering delirium, tremors, subsultus tendinum, petechial eruptions, and the patient sliding to the foot of the bed, that they are especially required. Nor is the property of the administration lessened by the tongue being dry and streaked with brown or black, and the teeth incrusted with sordes, provided attention be at the same time paid to the local affections, which these symptoms generally indicate, and measures be taken for their relief. Even when the collapse is not clearly indicated; when during the apparent favorable progress of the disease the pulse suddenly becomes soft and compressible, the skin cool and clammy, with a feeling of exhaustion, especially if there be a desire for wine, the necessity of employing some of the articles of this group of excitants is indicated. As far as regards quantity, from six to eight ounces of wine or a pint of weak punch, may be allowed in twenty-four hours at proper intervals." (p. 250.)

The following is part of Dr. Thomson's interesting account of the influence of mental excitants on the cure of diseases.

"With regard to the influence of mental excitants there can be no doubt, although their employment as therapeutical agents has been most unaccountably neglected. No man can practice his profession advantageously, however, who does not make himself acquainted with the anatomy of mind as well as that of the body: it is only such that can form an accurate conception of the influence of mental affections on the bodily frame, and how far not only moral happiness but corporeal health and vigour depend on the due application of mental energies. (p. 290)........Under many circumstances, joy has operated as a therapeutical agent. Alexander Trallianus has recorded a case of melancholia entirely cured by joy; and Corineous mentions an instance of a tertian being subdued by the same means. There are many instances of its curative influence in the writings of Hildanus and Etmuller. The conditions of the habit in which joy is most likely to display its salutary exciting influence are those of decided diminished action, such as occur in melancholia, hypochondriasis, and chlorosis. It may be demanded, how the highly pleasurable emotions are to be employed as remedial agents? Now, in reply, let me suppose that a medical practitioner is consulted for the relief of a dyspeptic affection, attended with hypochondriasis, which he can trace to moral affliction and disturbance in the nervous system. He finds that the feelings of his patient are quick, sensitive, and powerful, and perpetually harassed by the objects which surround him. His first step should be to remove him from these; and every means, the most powerful which can excite new impressions on the mind, should be adopted to overcome those which have caused and are keeping up the disease. The lively sports of the field, if the patient has any predilection for them; scenes of gaiety and animation; news of an agreeable kind; exhilarating conversation; and every exciting feeling of a pleasant description, must be courted and cherished." (p. 292.)

There is also sound observation in the following remarks; but we are not quite sure about the good dinner and the wine, which, although a temporary cure, might afterwards aggravate the disease.

"A very frequent disease connected with mental depression is nervous cephalalgia. The pains are generally acute over one, sometimes over both eyes. The brain in such a state is in an irritable, not in an inflammatory condition; hence stimulants are indicated, and we find that sometimes even those of a material kind, a good dinner and a glass of wine, will dissipate that which to a careless observer would have demanded cupping and other depleting measures. A much more immediate remedy, however, is any event which can cause pleasurable and cheerful feelings in the mind; under such the irritability and the headach will generally and instantly disappear."

In describing the effects of impetuosity, the author mentions the case of a gentleman in the last stage of phthisis, who died in consequence of excitement from this passion. (p. 294.) The same story is related at page 233, under the general article, "mental influences."

Our limits will not allow us to dwell longer on Dr. Thomson's work. In delivering our opinion as to its general character and merits, we may state that it is one of considerable merit, indicating very extensive research on the part of the author, and containing a large amount of valuable facts and information; but it contains much useless matter, and the style is often diffuse and the doctrines not unfrequently hypothetical or destitute of precision.

### III. Dr. Pereira's Materia Medica.

In his preface to this new edition of his work Dr. Pereira tells us, that "among the additions will be found articles on mental impressions, light, heat, cold, electricity, magnetism, diet, climate, and exercise, considered as therapeutic agents." Under the article "light" the author makes the following remarks on dioptric instruments, which we shall quote for the benefit of our weak-sighted readers:

"When vision is imperfect, from defect of focal distance, the remedy consists in the use of dioptric or refracting instruments (eye-glasses, spectacles.) In myopia (i. e. short or near-sightedness,) doubly-concave lenses (whose focal lengths vary from $2\frac{1}{2}$ to 48 inches,) are usually employed to counteract the over-refractive power of the humours; while in presbyopia (long or far-sightedness) doubly-convex lenses (whose focal lengths vary from about 6 to 48 inches) are generally used to obviate the diminished refractive power of the humours of the eye. Lenses for the above purposes are commonly made either of flint-glass or of Brazilian quartz. The latter, called pebble, has the advantage of greater hardness, and its surface therefore is not so readily scratched........

"*Chromatic instruments.* In some affections of the eye (popularly known as weakness of sight) coloured glasses are employed, with occasional relief, to diminish the intensity of the light. Those with a neutral tint (or twilight tinge) prove the most agreeable to the eye........Green, blue, indigo, and violet lights, are much less injurious than either red or yellow. Spectacles of these colours have been made for the use of those suffering with sensitive eyes, but they are inferior to the neutral tint before mentioned; since, after their removal from the eyes, every object sometimes presents for a short period complimentary tints, showing that these colours have fatigued the retina. All dark-coloured glasses, however, and especially black crape spectacles, are objectionable, on account of their greater power of absorbing and radiating caloric, by which they prove heating to the eyes." (pp 6-7, vol. i.)

The following are very judicious and useful remarks regarding the vapour-bath:

"As aqueous vapour, like air, is a worse conductor of caloric than liquid water, its influence, as a source of either heat or cold, is neither so powerfully nor so speedily felt as that of the latter. Hence, therefore, the temperature of the vapour-bath should always exceed that of the water-bath. If, however, the whole body be immersed in vapour, which is consequently inhaled, the temperature must be a little less than if the trunk and limbs alone were subjected to the influence of vapour; because the inhalation of vapour stops the cooling process of evaporation from the lungs. The following is a comparative view of the heating process of water and of vapour distinguishing the latter according as it is or is not breathed.

| | Water. | Vapour. | |
|---|---|---|---|
| | | Not breathed. | Breathed. |
| Tepid-bath ... ... ... | 85°— 92° | 96°—106° | 90°—100° |
| Warm-bath ... ... ... | 92 — 98 | 106 —120 | 100 —110 |
| Hot-bath ... ... ... | 98 —106 | 120 —160 | 110 —130 |

The vapour-bath is distinguished from the hot-air bath by its soothing, relaxing and greater sudorific influence; from the hot-water bath, by its inferior power of communicating heat, by its greater sudorific tendency, and by its causing scarcely any superficial compression of the body, whereby it does not occasion the precordial oppression experienced on entering the water-bath." (p. 15, vol. i.)

A good outline is given of the various substances used as food; but as the author has since published a distinct work on the subject, we shall have another opportunity of noticing his labours more at length. Besides the additions which have already been referred to, Dr. Pereira has improved the work by describing more in detail the process of the British pharmacopœias, "including those of the new Edinburgh one," and adding a large number of new woodcuts. On a former occasion, we expressed our high opinion of the first edition of this work. The present edition being enlarged and improved is necessarily still more worthy of approbation. As a record of materia medica, it is unrivalled in the combined qualities of fulness and accuracy by any other work in our language; although perhaps for the student and junior practitioner, one more condensed would be found better adapted for ordinary use. No one is so well qualified to write such a manual as Dr. Pereira.

